# Microstructural deficits of the thalamus in major depressive disorder

**DOI:** 10.1093/braincomms/fcac236

**Published:** 2022-09-17

**Authors:** Yuxuan Zhang, Yingli Zhang, Hui Ai, Nicholas T Van Dam, Long Qian, Gangqiang Hou, Pengfei Xu

**Affiliations:** Beijing Key Laboratory of Applied Experimental Psychology, National Demonstration Center for Experimental Psychology Education (BNU), Faculty of Psychology, Beijing Normal University, Beijing 100875, China; Department of Depressive Disorders, Shenzhen Kangning Hospital, Shenzhen Mental Health Center, Shenzhen 518020, China; Shenzhen Key Laboratory of Affective and Social Neuroscience, Magnetic Resonance Imaging Center, Center for Brain Disorders and Cognitive Sciences, Shenzhen University, Shenzhen 518052, China; Melbourne School of Psychological Sciences, The University of Melbourne, Melbourne 3010, Australia; MR Research, GE Healthcare, Beijing 100176, China; Department of Radiology, Shenzhen Kangning Hospital, Shenzhen Mental Health Center, Shenzhen 518020, China; Beijing Key Laboratory of Applied Experimental Psychology, National Demonstration Center for Experimental Psychology Education (BNU), Faculty of Psychology, Beijing Normal University, Beijing 100875, China; Center for Neuroimaging, Shenzhen Institute of Neuroscience, Shenzhen 518107, China

**Keywords:** depression, thalamus, structural connectivity, morphometry, quantitative MRI

## Abstract

Macroscopic structural abnormalities in the thalamus and thalamic circuits have been implicated in the neuropathology of major depressive disorder. However, cytoarchitectonic properties underlying these macroscopic abnormalities remain unknown. Here, we examined systematic deficits of brain architecture in depression, from structural brain network organization to microstructural properties. A multi-modal neuroimaging approach including diffusion, anatomical and quantitative MRI was used to examine structural-related alternations in 56 patients with depression compared with 35 age- and sex-matched controls. The seed-based probabilistic tractography showed multiple alterations of structural connectivity within a set of subcortical areas and their connections to cortical regions in patients with depression. These subcortical regions included the putamen, thalamus and caudate, which are predominantly involved in the limbic-cortical-striatal-pallidal-thalamic network. Structural connectivity was disrupted within and between large-scale networks, including the subcortical network, default-mode network and salience network. Consistently, morphometric measurements, including cortical thickness and voxel-based morphometry, showed widespread volume reductions of these key regions in patients with depression. A conjunction analysis identified common structural alternations of the left orbitofrontal cortex, left putamen, bilateral thalamus and right amygdala across macro-modalities. Importantly, the microstructural properties, longitudinal relaxation time of the left thalamus was increased and inversely correlated with its grey matter volume in patients with depression. Together, this work to date provides the first macro–micro neuroimaging evidence for the structural abnormalities of the thalamus in patients with depression, shedding light on the neuropathological disruptions of the limbic-cortical-striatal-pallidal-thalamic circuit in major depressive disorder. These findings have implications in understanding the abnormal changes of brain structures across the development of depression.

## Teaser

By using multi-modal neuroimaging techniques, Zhang *et al*. report that microstructural deficits of the thalamus, which associated with macroscopic abnormalities including disrupted structural connectivity and morphological abnormalities, characterize systematic deficits of brain architecture in major depressive disorder.

## Graphical Abstract

**Graphical Abstract:**
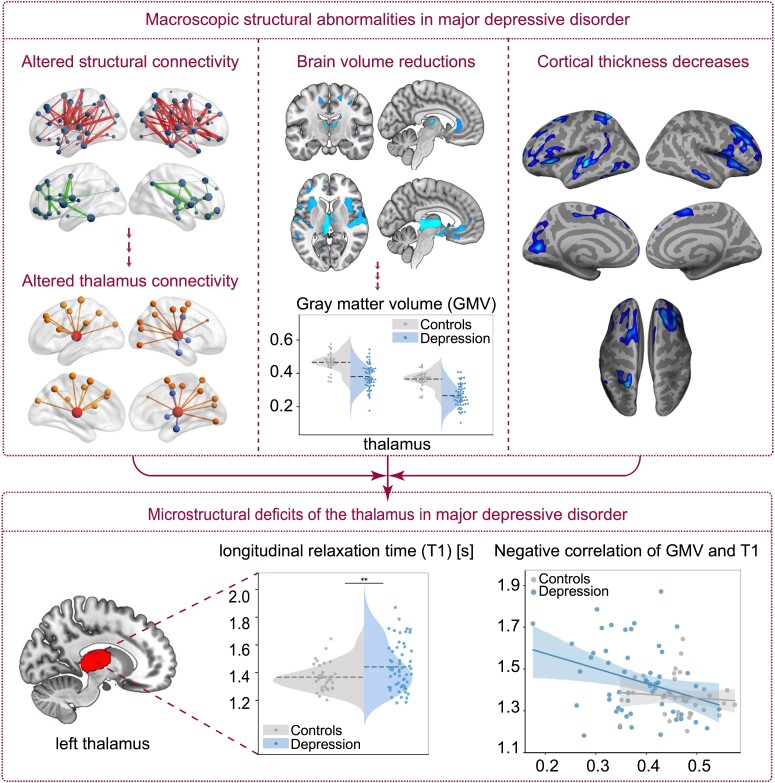

## Introduction

Major depressive disorder (MDD) is a globally prevalent and burdensome psychiatric illness associated with abnormal changes in several cognitive domains, including attention, memory and emotion processing.^1^ A large body of neuroimaging findings show structural deficits across multiple brain regions and widespread disruptions of connectivity between brain networks in MDD. These findings consistently converge on the limbic-cortical-striatal-pallidal-thalamic (LCSPT) circuit, which might underlie depressive pathology.^2–7^ In support of this hypothesis, morphometric abnormalities have been observed across all parts of the LCSPT circuit in MDD, including grey matter volume (GMV) and/or white matter volume (WMV) reduction in the thalamus,^8,9^ amygdala,^10^ caudate,^11^ putamen,^12^ nucleus accumbens^13^ and prefrontal cortex (PFC),^14^ as well as cortical thickness decreases in the orbitofrontal cortex^15^ (OFC). Complementary to the morphologic abnormalities, accumulating evidence has shown altered brain structural connectomes of key nodes within the LCSPT circuit in depression, involving decreased connectivity of white matter (WM) network and reduced grey matter (GM) structural covariance between the striatum, pallidum, thalamus and OFC.^16–20^

The thalamus is one of the key nodes within the LCSPT circuit. Anatomically interconnected with the PFC, striatum and amygdala, the thalamus serves as a hub in response to the relay and distribution of afferent signals.^2^ Its reciprocal connections with cortical and subcortical regions enable the thalamus to facilitate exchanges of subcortical information with the cortex.^21^ Neuroimaging findings have converged on thalamic involvement in macroscopic structural abnormalities in depression. Specifically, the thalamic volume reduction has been implicated in the pathophysiology of MDD and has been associated with severity of depressive symptoms.^22,23^ Diffusion tensor imaging (DTI) studies have shown abnormal structural connectivity and reduced WM tracts within the thalamo-frontal pathway in MDD.^17,18,24,25^ By combining DTI with graph-theoretic approaches, disrupted topological organizations in key regions of the thalamic network have also been shown in MDD.^26,27^ Collectively, these findings jointly converge upon structural abnormalities within the LCSPT circuit and its key nodes which may directly contribute to the neuropathology of MDD. Although macroscopic structural abnormalities in depression have been widely examined, quantitative alternations of cytoarchitectonic-related tissue properties underlying these macroscopic structural deficits in depression are not well understood.

By estimating brain macromolecular tissue volume (MTV) and longitudinal relaxation time (T1), the recently developed quantitative MRI (qMRI) technique can inform about macromolecular composition and organization.^28^ Importantly, brain macromolecular compositions and contents are accompanied by a set of microstructural processes related to tissue properties. For example, axons with myelination can be encased by the protective sheath, which has a high macromolecular content.^29^ These microstructural processes have been linked to macroscopic morphological changes of the brain.^30^ Given the sensitivity and specificity of qMRI parameters, such as MTV and T1, to microstructural tissue properties,^28,29,31^ we are able to quantify brain macromolecular composition *in vivo* and to make inferences about the underlying mechanism driving observed morphological findings. Specifically, MTV quantifies myelin volume on the basis that the brain macromolecules are principally cell membranes and proteins.^28^ T1 depends on both density of macromolecules and the local microenvironment, thus can be used as a marker of microstructural changes during brain development, such as dendrites, oligodendrocytes and myelination.^32^ Non-invasive brain tissue characterization using qMRI technique has significant implications for the identification of microstructural changes of specific regions and disease status monitoring in clinical practice.^28,32–37^ For instance, studies of patients with depression have reported significantly changes in qMRI-related parameters, such as lower longitudinal relaxation rate, R1 (1/T1), and increased proton density, which have provided empirical evidence for microstructural changes of brain tissue composition and myelin in depression.^36,37^ Therefore, the qMRI technique could be used to measure microscopic cytoarchitectonic-related properties underlying macroscopic structural alterations.

Here, we used a multi-modal neuroimaging approach comprising structural, diffusional and quantitative MRI to examine integrative structural abnormalities in patients with MDD, from macroscopic structural networks to the cytoarchitectonic-related microstructural level properties of the LCSPT circuit. We first explored abnormalities within- and across-network structural connectivity and the highly connected hub regions, as well as cortical thickness and subcortical volume across the whole brain. Next, we conducted a conjunction analysis to identify key regions that showed common structural alternations within the LCSPT circuit across multi-modal imaging data. Finally, we examined alternations in tissue properties as well as the relationship between tissue properties and macroscopic measurements of the identified key regions in MDD.

## Materials and methods

### Participants

In total, 56 MDD patients (aged 36 years, 41 females) and 35 age- and sex-matched healthy controls (HCs; aged 39 years, 22 females) were included in the present study. See Table 1 for details about demographic and clinical characteristics of all participants. For clinical details about the patients, see Supplementary Methods. All participants and/or the guardians of MDD patients had signed the informed consent before participating in the study. The study was approved by local ethics committee.

**Table 1:** Demographic and clinical characteristics of participants

	MDD (*n* = 56)	HC (*n* = 35)
Male sex, *n* (%)	15 (27)	13 (37)
Age, mean (SD), years	36.36 (15.96)	39.40 (14.77)
Educational level, mean (SD), years	12.82 (3.17)	14.94 (2.84)
HAMD score, mean (SD)	19.63 (7.61)	—
HAMA score, mean (SD)	16.23 (6.77)	—
Multiple depressive episodes, *n* (%)	22 (39)	—
Psychiatric comorbidities, *n* (%)	7 (13)	—
Current medications, *n* (%)	48 (86)	—
*Duration exposed to medications, n (%)*		
<3 months	24 (43)	—
≥3 months	24 (43)	—

HAMA, Hamilton anxiety rating scale; HAMD, Hamilton depression rating scale; HC, healthy control; MDD, major depressive disorder.

### MRI data acquisition

All diffusion, anatomical and quantitative MRI data were collected from all participants (*n* = 91) using a 3 T GE Discovery MR750 scanner (GE Medical Systems) at Shenzhen Kangning Hospital with an eight-channel head coil (see Supplementary Methods for details of scanning parameters).

### MRI data analysis

#### Diffusion MRI data analysis

**Figure 1:**
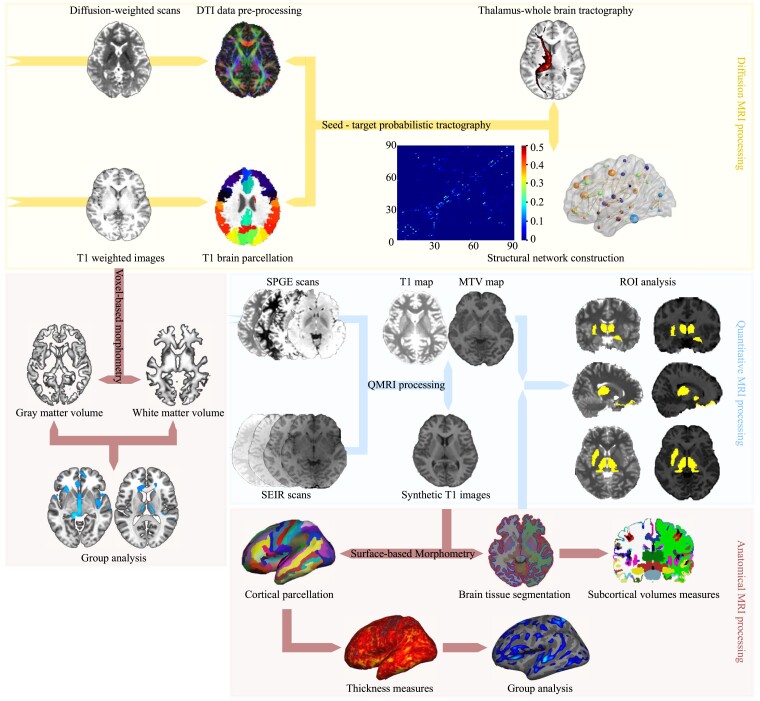
**An overview of the MRI data analysis workflow.** In diffusion MRI processing stream, the DTI model was constructed from the individual diffusion-weighted image. The T1-weighted brain images were parcellated into 90 cortical and subcortical regions according to the AAL atlas. Using ‘seed-target’ probabilistic tractography, the thalamus-specific connectivity and the whole-brain structural network were constructed. In quantitative MRI processing stream, the MTV and T1 maps with synthetic T1 images were generated and used for ROI analysis. In anatomical MRI processing stream, the synthetic T1 images were processed for the qMRI ROI analysis and SBM analysis. Additionally, individual T1-weighted images were processed for VBM analysis. DTI, diffusion tensor imaging; SPGE, spoiled gradient echo; SEIR, spin echo inversion recovery; MTV, macromolecular tissue volume; ROI, region of interest; SBM, surface-based morphometry; VBM, voxel-based morphometry.

The diffusion MRI data were first preprocessed followed by structural network construction and the network-based statistics (NBS) analysis^38^ to evaluate the whole-brain structural connectivity in MDD patients (Fig. 1). An automated anatomical labelling (AAL) atlas^39^ was used to parcellate the whole cerebral cortex into 90 regions/nodes (45 regions in each hemisphere, without the cerebellum; Supplementary Table 1) in native diffusion space, followed by probabilistic tractography between 90 AAL regions. This resulted in a connectivity matrix with inter-regional connection probability, representing a structural network for each participant. The NBS analysis was conducted to determine the difference of inter-regional structural connectivity between MDD and HC groups. Supra-threshold connections at a significant level of *P* < 0.01 with correction of family-wise error were reported. See Supplementary Methods for details.

For illustrating the connectivity patterns among large-scale brain networks in MDD patients, the identified increased and decreased structural connectivity were respectively categorized into an established seven-network parcellation of the human cerebral cortex with 100 parcels.^40^ A total of seven networks were defined based on the parcellation scheme, including the visual network, sensorimotor network, dorsal attention network, salience network (SN), limbic network (LN), frontoparietal network and default-mode network (DMN). Given the importance of subcortical regions in our study, we included the subcortical regions as an additional network division. Each AAL region involved in disrupted structural connectivity was localized into the seven networks based on its Montreal Neurological Institute (MNI) centroid coordinates.

#### Anatomical MRI data analysis

Voxel-based morphometry (VBM) analysis using the VBM8 toolbox (http://dbm.neuro.uni-jena.de/vbm8/) as well as surface-based morphometry (SBM) analysis using Freesurfer^41,42^ (http://surfer.nmr.mgh.harvard.edu) were performed to detect morphologic alternations in patients with MDD (Fig. 1; see Supplementary Methods).

#### Conjunction analysis

A conjunction analysis was conducted to identify regions that showed common alternations across all modalities, including structural connectivity, GMV/WMV and cortical thickness. Results from different analyses were integrated into AAL atlas based on the MNI coordinates of their peak voxel. The cortical regions were determined based on the consistent alternations across analyses of structural connectivity, VBM and SBM, while the subcortical regions were identified according to the analyses of structural connectivity and VBM. The identified regions, especially those play key roles within the LCSPT circuit, were included in subsequent qMRI analysis.

#### Quantitative MRI data analysis

The qMRI data were preprocessed by using the mrQ software package (https://github.com/mezera/mrQ) to produce the evaluation of MTV and quantitative T1 maps^28^ (Fig. 1). To examine microstructural disruptions underlying the common alterations in structural connectivity and regional morphology, we conducted region of interest (ROI) analyses on the qMRI measurements of the regions identified in the conjunction analysis (for details, see Supplementary Methods).

We conducted Pearson correlation analyses to examine the relationships between microstructural properties (MTV/T1) and two other measurements, the GMV and fractional anisotropy (FA) within key regions identified by conjunction analysis. Correlation analysis was conducted to test the influence of anxiety on microstructural alternations (see Supplementary Methods).

### Statistical analysis

Statistical analyses were performed using IBM SPSS (version 20). The χ^2^ test and *t*-test were conducted to compare potential between-group differences in gender, age and education, with a statistical significance of *P* < 0.05. For the volumes of subcortical regions, a general linear model was conducted controlling for overall brain volume and participants’ demographic variables (age, sex and education years) with a statistical significance of *P* < 0.05 after false discovery rate (FDR) correction. For ROI analysis of qMRI data, a two-sample *t*-test was conducted with participants’ age and gender as covariates to test group difference in MTV and T1 within each ROI. Significance was set at *P* < 0.05, FDR corrected.

### Data availability

The datasets reported in the current study are available upon request from the corresponding author. The data analysis scripts and codes generated and used in this work are available through the OSF (https://osf.io/6vpks/).

## Results

### Demographic and clinical characteristics

There were no significant differences in age nor gender between MDD patients and HCs (age: *t*_(89)_ = −0.910, *P* = 0.365; gender: χ^2^_(1)_ = 1.085, *P* = 0.353), but a significant group difference in years of education (*t*_(89)_ = −3.228, *P* = 0.002). We then repeated the MRI data analyses with and without the demographic data as covariates to test whether our results of MRI data analyses were affected by the demographic data. The results were robust after controlling for demographic variables (for details, see Supplementary Results).

### Alternations of whole-brain structural connectivity in MDD

**Figure 2:**
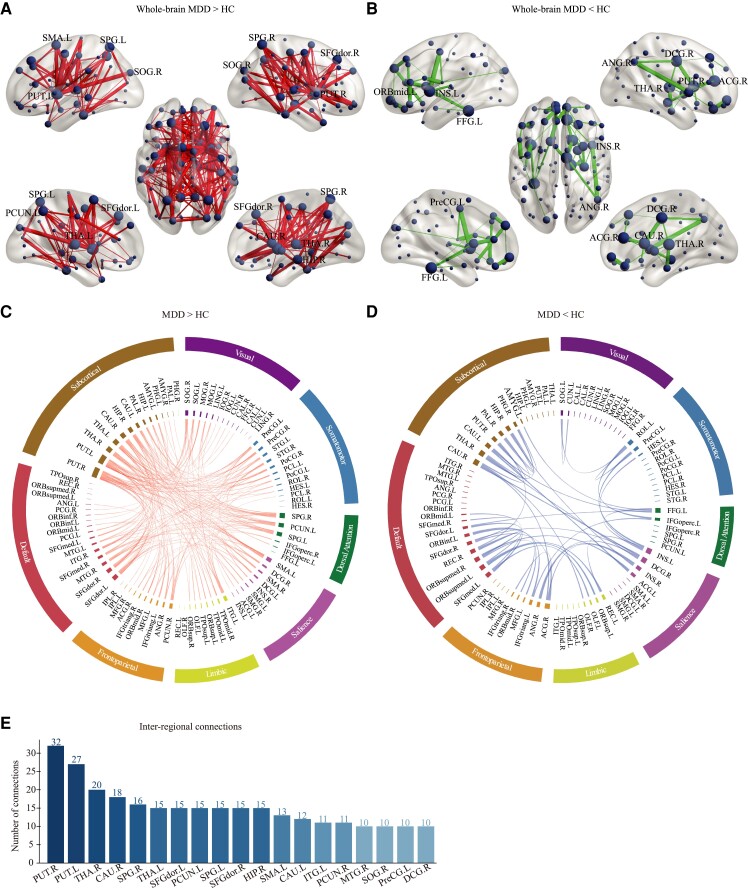
**Altered structural connectivity in MDD patients.** (**A**) Increased and (**B**) decreased connectivity identified by the NBS analysis in MDD patients relative to HCs. The node size indicates the number of edges connecting to the given node. Nodes with ≥10 increased connections or ≥4 decreased connections are labelled. The edge width represents the *t* value of connectivity between nodes. The circle plots depict (**C**) increased and (**D**) decreased connectivity patterns within and between seven networks and subcortical network. The AAL regions are represented by the inner segments of circle plots and are circularly arranged according to their number of connections. The angular size of each inner segment is proportional to the total connections of the given region. Networks are labelled on the outer segments. Regions within the same networks are shown in the same colour. The *t* values of connections are represented by ribbons between the inner segments. (**E**) The hub regions with ≥10 inter-regional connections (both increased and decreased) in the whole-brain structural connectivity are sorted in descending order. DA, dorsal attention; HCs, healthy controls; MDD, major depressive disorder. For abbreviations and indices of AAL regions, see Supplementary Table 1.

The NBS analysis showed two networks with disrupted structural connectivity in patients with MDD (Fig. 2). Specifically, one network comprised of 215 edges that connected to 76 nodes, showing significant increased structural connectivity in MDD (Fig. 2A, MDD > HC). The other network consisted of 44 edges linking to 38 nodes, showing significant decreased structural connectivity in MDD (Fig. 2B, MDD < HC).

At the large-scale networks level, we found that the most increased/decreased structural connections were those within the subcortical network, as well as those between the subcortical regions and cortical networks, including the DMN, SN and LN (Fig. 2C and D). Specifically, the increased structural connections within the subcortical network were observed between the HIP.R and left putamen (PUT.L; *t* = 3.56), the left thalamus (THA.L) and the right putamen (PUT.R; *t* = 3.39), the HIP.R and the left amygdala (AMYG.L; *t* = 3.32), the PUT.L and the right pallidum (PAL.R; *t* = 3.31), the AMYG.L and the left caudate (CAU.L; *t* = 3.26), the left hippocampus and the PUT.R (*t* = 3.31) and the HIP.R and the THA.L (*t* = 2.91). On the other hand, the decreased connectivity within the subcortical network included in the connections of the HIP.R with the PUT.R (*t* = 3.23) and PAL.R (*t* = 2.94), and the connections of the THA.R with the right parahippocampal gyrus (*t* = 3.64) and CAU.L (*t* = 3.45). With regard to the between-network connectivity, the increased between-network connectivity was expressed largely between the subcortical network and nodes of the DMN, specifically the THA.L and the left dorsolateral superior frontal gyrus (SFGdor.L; *t* = 3.24), the THA.L and the right dorsolateral superior frontal gyrus (SFGdor.R; *t* = 2.67), the THA.R and the SFGdor.L (*t* = 2.70), the CAU.R and the SFGdor.R (*t* = 3.38) and right medial superior frontal gyrus (*t* = 3.31), the PUT.R and the SFGdor.L (*t* = 3.88), SFGdor.R (*t* = 3.79) and right inferior orbitofrontal gyrus (*t* = 3.45) and between the HIP.R and the SFGdor.R (*t* = 2.74). The decreased between-network connectivity was mainly between the left superior medial orbitofrontal gyrus of the DMN and the left superior orbitofrontal gyrus of the LN (*t* = 4.18), and between the DCG.R of the SN and the CAU.R (*t* = 3.78) and THA.R (*t* = 2.88) of the subcortical network.

### Grey/white matter volume alternations in MDD

**Figure 3:**
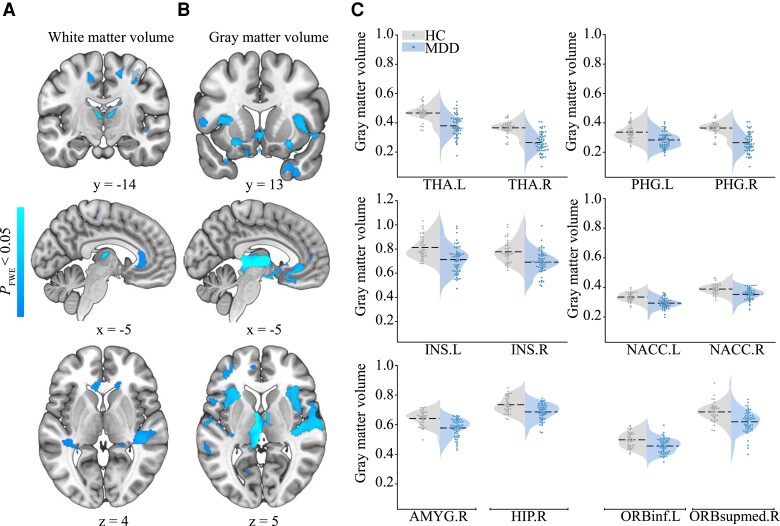
**Between-group differences in white matter volume and grey matter volume.** (**A**) The white matter volume (WMV) and (**B**) grey matter volume (GMV) reductions in MDD patients compared with HCs. The colour bar indicates *P*-value. Representative coronal, sagittal and axial slices of the supra-threshold clusters were overlaid on a MNI152 template. (**C**) The violin plots depict between-group differences of the GMV values within 12 key regions, where the width indicates subject density, the dashed line indicates average grey matter volume and the scatters indicate subject distribution for each group. Because the raw statistic map was transformed into TFCE values, only for the purpose of display, the GMV values of these regions were derived from the voxel-level statistic map obtained by using two-sample *t*-test with the threshold of *P* < 0.001 (uncorrected). HC, healthy control; MDD, major depressive disorder; NACC, nucleus accumbens. For abbreviations of AAL regions, see Supplementary Table 1.

Significant group differences were observed in both GMV and WMV (Fig. 3 and Table 2). The reduced GMV/WMV in MDD was not significantly influenced by the covariates (see Supplementary Results). There was no region showing larger GMV or WMV in MDD patients than in HCs.

**Table 2:** Clusters showed significant grey/white matter volume differences between MDD patients and HCs

Anatomical region^a^	Side	MNI coordinates^b^			Cluster size	*P-*value
		*x*	*y*	*z*		
*GMV*						
THA	L	−8	−6	4	1112	<0.001
AMYG	R	21	0	−15	353	0.001
HIP	R	18	−9	−15	265	0.001
PHG	L	−20	−26	−18	516	0.002
PHG	R	14	0	−18	407	0.002
THA	R	10	−9	7	328	0.005
STG	R	65	−20	12	2480	0.005
INS	L	−32	24	0	1345	0.005
HES	R	39	−24	9	528	0.005
ORBinf	L	−21	18	−14	583	0.006
PUT	L	−24	17	6	187	0.006
REC	R	2	51	−18	551	0.007
OLF	L	−20	6	−12	391	0.007
SMG	R	56	−33	27	672	0.008
INS	R	39	11	2	2259	0.009
REC	L	0	49	−18	925	0.009
ORBsupmed	R	9	51	−12	768	0.009
ACG	L	−4	35	−9	423	0.009
MTG	R	66	−22	−3	160	0.010
STG	L	−60	−17	9	1731	0.017
MFG	L	−33	50	1	280	0.017
HES	L	−51	−16	10	299	0.018
TPOmid	R	21	12	−36	522	0.019
ORBmid	L	−39	45	0	193	0.019
FFG	R	28	5	−41	687	0.021
SFGdor	L	−16	50	34	808	0.027
MTG	L	−63	−33	2	382	0.035
*WMV*						
THA	R	10	−22	9	613	0.004
THA	L	−6	−15	10	431	0.006
PreCG	R	32	−19	57	1192	0.010
PreCG	L	−28	−22	55	625	0.015
PoCG	L	−26	−40	48	608	0.017
SMA	R	9	−27	58	446	0.017
PCL	R	6	−30	61	330	0.018
PCUN	L	−15	−42	60	244	0.018
DCG	L	−14	−16	45	214	0.021
CAU	R	16	−18	21	397	0.022
IFGoperc	R	36	8	30	128	0.029
SFGdor	R	15	11	54	302	0.030
PoCG	R	30	−27	60	217	0.031
ACG	L	−3	35	−2	180	0.031
STG	R	56	−24	1	862	0.034

GMV, grey matter volume; HC, healthy control; L, left; MDD, major depressive disorder; R, right; WMV, white matter volume.

^a^ For full names of these abbreviations from AAL atlas, see Supplementary Table 1.

^b^ The MNI coordinates of the voxel with maximum statistical significance for identified clusters. Each cluster was represented by a coordinate of the peak voxel in this cluster.

### Cortical thickness and subcortical volume alternations in MDD

**Figure 4:**
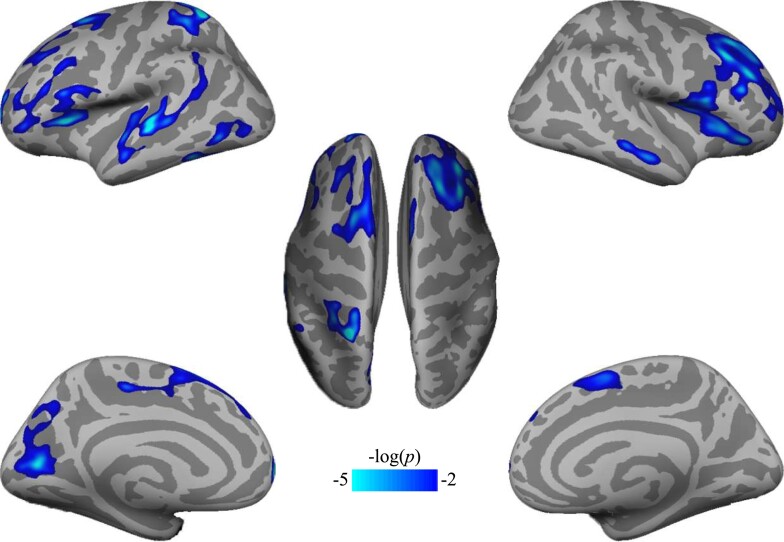
**Cortical thickness decreases in MDD patients compared with HCs.** Significant differences in cortex thickness were mapped onto the bilateral inflated cortical surfaces at the lateral and medial views. The colour bar represents *P-*value. L, left hemisphere; R, right hemisphere.

We found 14 clusters where cortical thickness was significantly lower in MDD patients relative to HCs (Fig. 4 and Table 3). No significant increases in cortical thickness were detected in the MDD group. None of the subcortical volumes showed significant group differences with FDR correction.

**Table 3:** Surface-based cluster summary of significant differences in cortex thickness between MDD patients and HCs

Anatomical location^a^	Side	Size (mm^2^)	MNI coordinates^b^			CWP
			*x*	*y*	*z*	
MFG	R	3790.43	37.1	27.3	39.9	0.0002
MTG	L	2277.56	−62.2	−21.5	1.8	0.0002
ORBinf	R	2141.24	43.5	33.5	−13.3	0.0002
SFGdor	L	1739.25	−19.5	10.3	56.0	0.0002
CAL	L	1696.76	−15.4	−77.3	10.9	0.0002
SPG	L	1170.05	−25.5	−54.8	63.6	0.0002
INS	L	1163.41	−31.9	26.5	9.0	0.0002
ORBmid	L	1138.21	−38.4	44.7	−3.8	0.0002
SFGmed	L	667.90	−11.7	63.0	2.5	0.0024
ITG	L	529.68	−49.8	−62.8	−5.9	0.014
ITG	L	502.62	−50.3	−48.8	−17.2	0.019
MFG	L	488.18	−25.7	28.4	33.5	0.023
SMA	R	481.17	7.4	4.5	59.7	0.030
MTG	R	471.28	60.0	−15.6	−16.8	0.034

CWP, cluster-wise *P-*value; HC, healthy control; L, left; MDD, major depressive disorder; R, right.

^a^ For full names of these abbreviations from AAL atlas, see Supplementary Table 1.

^b^ Coordinates of the peak vertex for this cluster.

### Cross-modality structural alternations in MDD

The conjunction analysis identified five regions with common cross-modal structural alternations. Specifically, there were one cortical, the left middle orbitofrontal gyrus, and four subcortical regions, the left putamen, right amygdala and bilateral thalamus, involved across structural connectivity and morphologic features (Supplementary Table 2).

### Microstructural alternations of the thalamus in MDD

**Figure 5:**
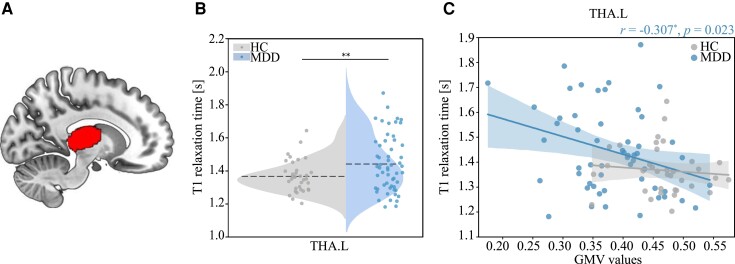
**Group differences in T1 values and the correlation with grey matter volume.** (**A**) The volumetric mask of the left thalamus was mapped on a MNI152 template for visualization. (**B**) The violin plot shows between*-*group differences in T1s in the left thalamus using two-sample *t*-test (*t*_(89)_ = 2.687, *P* = 0.009). (**C**) Correlation between grey matter volume and T1 values in the left thalamus in MDD group. The correlation coefficient (*r*) and *P*-value in MDD group were shown on the upper right of the plot. Note that the left thalamus was segmented based on Destrieux Atlas in individual space. GMV, grey matter volume; HC, healthy control; MDD, major depressive disorder; THA.L, the left thalamus.

*MTV/T1 alternations.* We found the averaged T1 values were significantly higher in MDD patients than in HCs in the left thalamus (THA.L; *t*_(89)_ = 2.687, *P* = 0.009; Fig. 5B). This result was not significantly correlated with levels of education (*r* = −0.193, *P* = 0.067) or anxiety (*r* = −0.259, *P* = 0.127). No significant between-group difference in qMRI measures was observed in other ROIs after FDR correction.

*Relationships of microstructural changes with macroscopic alternations.* The correlation analysis of qMRI measurements showed that T1s were inversely correlated with the GMV values in the THA.L (*r* = −0.307, *P* = 0.023; Fig. 5C) specifically in MDD but not in HCs. There was no significant correlation observed between T1s and FA values in the THA.L in MDD group (*r* = −0.109, *P* = 0.423).

## Discussion

To our knowledge, this is the first study combining macro–micro neuroimaging techniques to characterize structural abnormalities of the neural pathway that underlies the neuropathology of MDD. Three main findings are revealed in the present study. First, subcortical regions involved in neural circuits of MDD exhibit disrupted within- and between-network connectivity. Second, hub regions that are highly interconnected in structural networks consistently show morphological abnormalities. Third, the thalamus of the LCSPT circuit exhibits both across modal macrostructural alternations and cytoarchitectonic-related abnormalities in MDD. These findings elucidate the primary role of the thalamus in macro-to-micro structural abnormalities of depression.

### Disrupted structural brain networks in MDD

We found that patients with MDD were characterized by abnormal structural connectivity within subcortical network and between the subcortical network and DMN/SN. Consistent with previous findings,^24,26,27^ these results suggest the centric roles of the subcortical areas in disrupted brain connectivity of MDD. Importantly, these subcortical structures, including the bilateral thalamus, bilateral putamen and right caudate, are highly connected, responsible for the normal regulation of mood and involved in a variety of neural circuits of MDD.^2,3^ Evidence from neuroimaging and neuropathological studies suggests that abnormalities within and between these structures potentially account for disturbances in emotional behaviour and other aspects of depressive syndromes.^43,44^ For example, disrupted WM connectivity within the frontal-subcortical networks,^24^ as well as decreased WM tracts in the frontothalamic loops have been found in depressed patients.^25^ These hub regions form the LCSPT circuit, which is known for its central involvement in mood disorders like depression.^2–4^

Of this circuit, the thalamus is critical for cognitive and emotional processes through its higher order relay. Among the higher order thalamic relay nuclei, the mediodorsal thalamic nucleus has reciprocal connections with the PFC, relaying substantial subcortical projections from the amygdala and parahippocampal cortex to the orbital and medial PFC.^2,4^ Given its broad connections with cortical and subcortical structures, growing evidence suggests mapping thalamic connectivity can help to characterize distributed circuits abnormalities in neuropsychiatric disorders.^45^ In this context, our findings show increased connections of the thalamus with the PFC, bilateral putamen, caudate and pallidum in depression, which are key components of the LCSPT circuit. These findings provide multi-modal neuroimaging evidence for the neuropathology of MDD, where the thalamus plays a central role, connecting from prefrontal top–down control regions to the amygdala and putamen that responsible for the bottom–up processing of emotional signals.

At the network level, a lot of evidence has shown abnormalities in functional connectivity within the DMN and SN in depression.^46,47^ By synthesizing these findings, a triple network model has been proposed to particularly emphasizes the roles of the DMN and SN in abnormal patterns of functional connectivity in the depressed brain.^48^ Our findings revealed disrupted structural connectivity within the DMN and SN in MDD patients. Specifically, the precuneus of DMN and the insula of SN were involved in most of the abnormal connections with nodes of subcortical network, e.g. the putamen, thalamus, caudate and hippocampus. We suggest that these key nodes represent structural hubs particularly sensitive to network-level disruptions in MDD. Notably, the structural hubs we identified are also known to be functionally altered in depression based on previous evidence of functional connectivity.^49^ Taken together, these findings support the triple network model in depression by providing evidence of both structural and functional connectivity.

### Abnormal morphometry alternations in MDD

The hub regions altered in network connectivity also showed abnormal morphologic alterations, with specific volumetric losses and thickness decreases. Volume losses in subcortical structures of the LCSPT circuit have been associated with the pathology of MDD,^44^ and have been proposed as potential markers of MDD.^50^ For example, the reduced thalamus volume has been observed in the first-episode, untreated MDD patients,^9^ which was thought to help account for its maladaptive bottom–up processing of negative stimuli in MDD, and therefore is considered to be a potential marker of MDD.^23^ A recent meta-analysis has shown the corpus striatum, which comprises the caudate and putamen, was smaller in MDD.^10^ Especially, volume reduction of the caudate has been more extensively studied in depression and has shown to be associated with suicidality.^51^ Our findings showed both the GMV and WMV of the bilateral thalamus and GMV of the left putamen were reduced in MDD patients, which confirm the morphologic abnormalities of the thalamus and striatum in MDD. Crucially, our results showed consistently morphologic abnormalities and disrupted structural connectivity of the bilateral thalamus, right hippocampus and left putamen, suggesting the dysconnectivity within the LCSPT circuit in MDD. Besides, we also observed disrupted structural connectivity and decreased GMV and thickness of the left insula, one of key regions in the detection of ‘salient’, suggesting structural deficits of the insula also contribute to the neuropathology of MDD.

Numerous studies have shown widespread GMV/WMV reductions in MDD, especially the hippocampus, which is one of the most predominant regions that consistently showing volume reduction,^10,52^ suggesting hippocampal abnormality is a contributor to the pathogenesis of depression. Previous studies have also shown volume reductions as well as cortical thinning of the PFC and OFC in MDD.^14,53,54^ Consistently, we found reduced GMV of the right hippocampus, reduced PFC and OFC volume and decreased OFC thickness in bilateral hemispheres, suggesting critical roles of these brain regions in the neuropathology of depression. Taken together, our findings provide multi-modal neuroimaging evidence for the structural abnormalities of the LCSPT circuit in MDD. These findings are informative with regard to the neurobiological mechanisms that underlying cognitive and affective impairments in MDD.

### Abnormal microstructural alternations of the thalamus in MDD

Our study has revealed for the first time that cytoarchitectonic-related properties of the thalamus were altered in the depressive brain, while the thalamus was also involved in macroscopic structural abnormalities including disrupted structural connectivity and volume reductions in MDD patients. As aforementioned, we observed that a set of ‘key regions’ were involved in common structural abnormalities in MDD across multi-modalities. These key regions formed the LCSPT circuit, which is associated with depressive symptoms,^3,4,55,56^ as well as emotional and cognitive deficits in MDD.^5,57,58^ For example, the structural abnormalities of the thalamus such as volume loss are thought to be associated with its dysfunction in MDD.^23^

Given T1 decrease has been associated with the developmental change of brain tissue,^32^ larger T1 of the left thalamus in our study reflects abnormal development of the thalamus in MDD patients. Because T1 is sensitive to both MTV and tissue composition,^32^ larger T1 could be driven by multiple factors associated with tissue development, such as microstructural proliferation and myelination.^59^ The myelin volume which increases within the cortex across development is a likely source of T1 changes. While the T1 in both white and grey matter is attributable primarily to myelin, it is also influenced by water content,^30,60^ iron concentration^61,62^ and macromolecular composition.^28^ Future studies are necessary to examine the contributions to T1 abnormality of brain tissues in MDD.

Importantly, we found a negative correlation between T1 and GMV values of the left thalamus in MDD patients, which provides critical information for microstructural basis of macrostructural deficits in depression. Given the T1 is associated with multiple tissue characteristics,^28^ the observed negative correlation between T1 and GMV indicates that the macrostructural morphologic alterations are related to macromolecular composition and local microenvironment of the left thalamus in MDD patients. Previous findings have shown development could create new tissue that displaces water, resulting in higher MTV and FA.^59^ However, we did not observe any correlation between MTV and FA of the left thalamus, suggesting the microstructural alternations of the left thalamus may result from other biological factors such as axon diameter, axon density and myelin-sheath thickness.^63^ Collectively, the microstructural properties of the thalamus are closely associated with its macroscopically structural abnormalities in MDD, providing microstructural evidence for structural abnormalities in depression.

Several limitations of the present study should be noted. As we did not collect the information of medication use, we cannot make strong inferences about how macroscopic and microstructural alternations of the thalamus might be affected by medication use. Future studies are necessary to replicate the findings across the first-episode, unmedicated patients. In addition, although our results were largely unchanged after controlling for participants’ demographic variables, the MDD group and HCs were not matched for years of education. Despite that the methodology used in our study was relatively consistent and reliable, the combination of these methods in multi-modal MRI data analysis would weaken test-retest reliability due to the inherent limitations of specific methods. For example, probabilistic tractography can lead to unrealistic fibre tracts without anatomic justification, whereas morphometry measurements may be affected by analysis parameters. Although we used reliable methods for multi-modal analyses, corrected for multiple comparisons and controlled for covariate, the relatively small sample of the current study could also result in false positives of large effect sizes. Our findings need to be replicated with larger samples in future studies. Another limitation may come from the atlas we used in brain parcellation. Previous studies have shown unique advantage of specific parcellation/atlas for different modalities.^64,65^ In line with previous studies, to make the optimal measure of each modality, we used Freesurfer built-in atlas and AAL atlas for subcortical volume measures and tractography analyses, respectively. Because the node definition by different parcellations could produce different connectivity patterns of brain networks,^66^ the atlas used in the present study may have specific effects on the alternation of structural connectivity in MDD. Future studies can validate the consistency of structural connectivity across different parcellation schemes. The current study only focused on the microstructural alternations in specific regions associated with cross-modal macroscopic abnormalities, which restricted the exploration for whole-brain-wise microstructural features in MDD. Future studies can quantify qMRI measures across the whole brain and explore how microstructural properties altered in depression. Finally, the sample size of MDD patients and HCs were different in the current study, however, both MDD and HC samples were approximately normal distributed with the same variance. Therefore, the *t*-test we used in statistical analyses is applicable and reliable for group comparisons of different sample sizes.^67^

## Conclusion

By using multi-modal neuroimaging techniques, we show consistently structural brain abnormalities in depression, from network connectivity, morphometric volume and thickness, to cytoarchitectonic-related properties. The LCSPT circuit plays a centric role in the structural brain alterations of depression, of which the thalamus is indispensable, with microstructural deficits underlying the macroscopic morphological changes. Our study sheds lights on the understanding of neuropathology of MDD and has important implications in the diagnosis and treatment of depression.

## Supplementary Material

fcac236_Supplementary_Data

## Abbreviations

AAL =: automated anatomical labelling
DMN =: default-mode network
DTI =: diffusion tensor imaging
FA =: fractional anisotropy
FDR =: false discovery rate
GM =: grey matter
GMV =: grey matter volume
HC =: healthy control
LCSPT =: limbic-cortical-striatal-pallidal-thalamic
LN =: limbic network
MDD =: major depressive disorder
MNI =: Montreal Neurological Institute
MTV =: macromolecular tissue volume
NBS =: network-based statistic
OFC =: orbitofrontal cortex
PFC =: prefrontal cortex
qMRI =: quantitative MRI
ROI =: region of interest
SBM =: surface-based morphometry
SN =: salience network
T1 =: longitudinal relaxation time
VBM =: voxel-based morphometry
WM =: white matter
WMV =: white matter volume
