# Supplementary material for: Microstructural deficits of the thalamus in major depressive disorder
Source: Brain Commun. 2022 Sep 17;4(5):fcac236. doi: 10.1093/braincomms/fcac236 (PMC9525011; doi:10.1093/braincomms/fcac236)
Supplement: fcac236_Supplementary_Data [file fcac236_supplementary_data.pdf]

Microstructural deficits of the thalamus in major depressive disorder

Supplementary Materials

Supplementary Methods 1
Participants 1
MRI data acquisition 1
Diffusion MRI data analysis 2
Data preprocessing 2
Network node definition 2
Network construction 2
NBS analysis 3
Structural connectivity analysis of the thalamus with the whole brain. 3
Anatomical MRI data analysis 4
Voxel-based morphometry analysis 4
Surface-based morphometry analysis. 4
Quantitative MRI data analysis 5
Data preprocessing 5
ROI analysis for qMRI data. 5
Correlation analysis of qMRI measurements. 5
Supplementary structural network analysis 6
Supplementary VBM analysis 6
Supplementary Results 6
Alternations of the thalamus-centered structural connectivity in MDD patients 6
The correlation of qMRI measurements across 2 groups 7
Results of supplementary structural network analysis 7
Results of supplementary VBM analysis 7
Supplementary Reference 9
Supplementary Table 1. Abbreviations of regions from AAL atlas. 10
Supplementary Table 2. Regions with cross-modality structural alternations in MDD patients. 12
Supplementary Table 3. Clusters showed significant gray matter volume/white matter volume differences between MDD patients and HCs after controlling for the demographic data. 12
Supplementary Figure 1. Significant altered structural connectivity and connectivity patterns in the thalamus-centered network. 14
Supplementary Figure 2. Correlation between T1 and gray matter volume values in the left thalamus across 2 groups. 15
Supplementary Figure 3. Altered structural connectivity in MDD patients with all covariates removed. 16
Supplementary Figure 4. Between-group differences in gray and white matter volume with all covariates removed. 18

Supplementary Methods

Participants

MDD patients were recruited from Shenzhen Kangning Hospital, diagnosed based on the Structured Clinical Interview for Diagnostic and Statistical Manual of Mental Disorders, Fifth Edition (DSM-V) criteria, and met the following criteria: 1) age range of 18 to 65 years; 2) for male/female who is capable of fertility, s/he agreed to use effective contraceptive methods during the study period and within one month after the end of the study to ensure the effective contraception for himself/herself or sexual partner. The exclusion criteria were as follows: 1) participants met the DSM-V diagnostic criteria for another psychiatric illness, including schizophrenia, bipolar disorder, anxiety disorders, obsessive-compulsive disorder, physical symptoms, organic mental disorders or depression caused by hypothyroidism, substance abuse or dependence; 2) participants who have received electroconvulsive therapy (ECT) within three months before screening; 3) participants with any other unsuited conditions considered by investigators that could not participate the study. The severity of depressive symptoms was assessed using the Hamilton Depression Rating Scale (HAMD). Because of the high comorbidity of depression and anxiety[1], we assessed the severity of anxiety for MDD patients using the Hamilton Anxiety Rating Scale (HAMA) and examined the influence of anxiety in subsequent analyses. All HCs were cognitively normal, free of neurological disease, and had no cognitive complaints. For MRI data quality checks, data of one HC were excluded from diffusion MRI (dMRI) data analysis due to the pool image quality, resulting in 56 MDD patients and 34 HCs included in the dMRI data analysis.

MRI data acquisition

High-resolution structural images were acquired with a three-dimensional (3D) T1-weighted (T1w) fast spoiled gradient-echo (FSPGR) sequence with TE = 2.9 ms, TR = 6.7 ms, FA = 12°, field of view (FoV) = 256 × 256 mm, 192 slices, and voxel size = 1 mm^3. The dMRI data was collected using a single-shot spin-echo echo-planar imaging (EPI) sequence (TR = 8724 ms, TE = 81.4 ms, FA = 90°, voxel size = 2 × 2 × 2 mm^3). The diffusion weighting was isotropically distributed along 64 directions (b = 1000 s/mm^2). Ten non-diffusion weighted (b = 0) images were acquired at the beginning of the dMRI session. The quantitative MRI (qMRI) data was collected using the protocols described in a previous study[2]. In brief, the quantitative macromolecular tissue volume (MTV) and longitudinal relaxation time (T1) were measured from four spoiled gradient echo (SPGE) images with flip angles of 4°, 10°, 20°, 30°(TR = 14 ms, TE = 2 ms) at a 1 × 1 mm^2 in-plane resolution with a slice thickness of 2 mm. We collected four additional spin-echo inversion recovery (SEIR) scans with an EPI read-out, a slab inversion pulse, and spectral spatial fat suppression to remove field inhomogeneities. The SEIRs were acquired with a TR of 3 s, echo time set to minimum full, and 2x acceleration. The inversion times were 50, 400, 1200, and 2400 ms with a 2 × 2 mm^2 in-plane resolution and a slice thickness of 4 mm.

Diffusion MRI data analysis

Data preprocessing

The dMRI data were preprocessed using the FMRIB's Diffusion Toolbox (FDT) from FSL package[3,4] (version 5.0.9; https://fsl.fmrib.ox.ac.uk/fsl). Briefly, diffusion-weighted images were first corrected for eddy-current-induced distortions and head movements by using affine transformation of each diffusion-weighted image to non-diffusion-weighted (b = 0) image. The diffusion tensor models were then fitted at each voxel of the brain on distortion-corrected images to yield three eigenvectors and three eigenvalues.

Network node definition

To determine the nodes of structural network, an automated anatomical labeling (AAL) atlas was used to parcellate the whole cerebral cortex into 90 regions/nodes (Supplementary Table 1). The mask for each brain region was extracted and transformed into each participant's diffusion space. In brief, ac-pc aligned T1w image in individual participant' s space was first normalized to the ICBM 152 template in Montreal Neurological Institute (MNI) space by applying nonlinear transformation between T1w structural space and standard space. Second, the T1w image was coregistered to participant' s non-diffusion-weighted (b = 0) image by using linear transformation between diffusion space and the T1w structural space. Here, relevant transformation matrices and their inverses were derived and concatenated to produce transformation matrices between diffusion and standard space. The concatenated transformation matrices were then used to warp AAL labels from MNI space to native diffusion space. This allowed us to perform inter-subject comparison in diffusion space and produce an individual-specific connectivity matrix. To this end, 90 parcellated regions for each participant in native diffusion space were produced, representing 90 nodes in the structural network, and used for structural network construction.

Network construction

To reconstruct the structural network of white matter tracts between 90 AAL regions, probabilistic tractography was performed on the preprocessed dMRI dataset using PANDA pipeline[5] (http://www.nitrc.org/projects/panda) in MATLAB2018b. We used a within-voxel probabilistic diffusion model to build up distributions on diffusion parameters at each voxel of the dataset[6]. The probability distribution estimates the multi-fiber tract orientation as well as its uncertainty in each voxel and guides multiple fiber samples starting from a seed voxel to a specified target region. Probabilistic tractography was then applied by drawing 5000 individual streamlines (maximum step: 2000, step length: 0.5mm, curvature threshold: 0.2) in each voxel in native diffusion space. Structural connectivity between two regions was measured by calculating connectivity probability from each seed region to each of the other target regions. In the present study, each AAL region was selected as the seed and the remaining 89 regions as targets. The tracts were terminated as soon as leaving the brain or reaching a particular target region. This resulted in a 90×90 connectivity matrix with inter-regional connection probability, representing a structural network for each participant. For this matrix, the probability from the i-th region to the j-th region is not necessarily equivalent to the one from j to i. However, these two probabilities are highly correlated across the cerebral cortex. Thus, a unidirectional

symmetric matrix for each participant was acquired by averaging these two probabilities. In native diffusion space, the resulted structural network is composed of nodes, representing a pair of regions, and edges, representing structural connectivity among the nodes, i.e., the region (i) and another region (j) were connected through an edge (e_{ij} = [i, j]), in case of at least one fiber existed between them[7,8]. Consequently, given the definition of nodes and edges for the structural connectivity network (matrix), the connectivity probability was calculated as an edge between pair of regions[6,9–11].

NBS analysis

The NBS approach was applied for group comparisons by using NBS Connectome (http://www.nitrc.org/projects/nbs/). The NBS is a non-parametric statistical method to deal with the multiple comparisons problems by controlling for the FWE rate in a weak sense while performing mass univariate hypothesis testing on all connections. With the NBS, the null hypothesis was evaluated at a level of interconnected subnetworks rather than individual connections. Briefly, a two-sample *t*-test at each edge was conducted independently to test for significant differences in the value of connectivity between the two groups. A primary threshold ($t \geq 2.5$, which corresponds to uncorrected $p \leq 0.01$) was then applied to define a set of supra-threshold connections. This step identified all the possible connected components in the matrix at the uncorrected level, followed by computing the size of the connected components which link supra-threshold connections with a set of nodes in the network. To estimate the statistical significance of size for each component, the null distribution of maximal connected component size was empirically derived using non-parametric permutation testing with 5000 times. Supra-threshold connections that were significant at the level of $p < 0.01$ were reported. The BrainNet viewer (http://www.nitrc.org/projects/bnv/) was used to visualize and display the significant connections[12].

Structural connectivity analysis of the thalamus with the whole brain.

A seed-based probabilistic tractography was conducted to evaluate white matter structural connectivity of the thalamus with the whole brain in patients with MDD. The steps followed similar procedures with the seed-target probabilistic tractography as described above (see **Network construction**) but only each unilateral thalamus was defined as a seed and voxels of the rest of the whole brain were set as targets. The unilateral thalamus seed mask adapted for the analysis was derived from the individual T1 parcellation based on AAL atlas. A two-sample *t*-test was conducted to compare the thalamus-centered structural connectivity between MDD patients and HCs. The multiple comparison correction was performed using threshold-free cluster enhancement (TFCE), a non-parametric method to enhance detectability of neuroimaging signal on cluster-based thresholding by integrating information of cluster extend and height into voxel-wise statistical inference[13]. The TFCE-based analysis was performed with 5000 permutation tests and the resulting TFCE-statistic map was considered significant at cluster-wise $p < 0.05$, FWE-corrected.

Anatomical MRI data analysis

Voxel-based morphometry analysis

To detect macroscopic abnormalities of brain volumes in MDD, voxel-based morphometry (VBM) analysis was performed using VBM8 toolbox (http://dbm.neuro.uni-jena.de/vbm8/) implemented in SPM 12 (Statistical Parametric Mapping, http://www.fil.ion.ucl.ac.uk/spm/software/spm12) software running on MATLAB R2018 for Linux. All ac-pc aligned T1w images were segmented into the gray matter (GM), white matter (WM) and cerebrospinal fluid (CSF) in native space. The segmented images were then normalized to MNI space with a dimension of $121 \times 145 \times 121$ and a spatial resolution of $1.5 \times 1.5 \times 1.5$ mm using high-dimensional DARTEL normalization. The normalized segmented images for GM and WM were subsequently modulated by Jacobian determinants derived from the spatial normalization, which allows to estimate the GM volume (GMV) and WM volume (WMV) while controlling for overall brain size. After preprocessing, sample homogeneity was checked to identify outliers which were two standard deviations outside of the mean. Finally, these normalized, segmented, modulated volumes were spatially smoothed with a 6-mm full-width half maximum (FWHM) Gaussian kernel. To avoid possible edge effects around the margin between different tissue types, all voxels with a probability value < 0.2 (absolute threshold; range 0-1) for both GM and WM were excluded. For statistical analysis, a two-sample *t*-test was conducted to identify significant differences in GMV and WMV between MDD patients and HCs, respectively, and the TFCE method with 5000 permutation tests was used for correcting the multiple comparisons. The resulted TFCE-statistic map was thresholded at $p < 0.05$, FWE-corrected.

Surface-based morphometry analysis.

The synthetic T1w images generated from mrQ analysis pipeline were then processed for surface-based morphometry (SBM) analysis by using Freesurfer v5.3.0 (http://surfer.nmr.mgh.harvard.edu) with standard recon-all procedures. The Freesurfer pipeline performs the volumetric GM and WM segmentation, providing several automated cortical parcellations and assigning a neuroanatomical label to each location based on prior atlas. The processing steps included skull stripping, atlas registration, spherical surface registration and parcellation. In this study, cortical parcellation and subcortical segmentation were based on the Freesurfer built-in atlas[14,15], resulting in a total of 82 distinct brain regions, including 74 cortical, 7 subcortical regions (the thalamus, caudate, putamen, pallidum, hippocampus, amygdala and nucleus accumbens) per hemisphere and the brainstem. The resulted segmented brain tissues were used for subsequent region of interest (ROI) analysis of qMRI modality. After the recon-all procedures of all participants, cortical thickness maps were smoothed using Gaussian Kernel with FWHM of 10 mm. The vertex-wise group comparison for cortical thickness was performed in Freesurfer using general linear model (GLM) with age, gender and years of education as covariates. Moreover, the vertex-wise results were Bonferroni corrected for multiple comparisons with a threshold of 2, corresponding to $p < 0.01$. Cluster-wise statistical threshold of $p < 0.05$ was used after Bonferroni correction. A statistical thickness difference map was constructed using -log10 (*p* value). Cluster-wise *p* values (CWP) were reported. The volumes of subcortical regions were automatically derived from Freesurfer's output report for group comparisons.

Quantitative MRI data analysis

Data preprocessing

Both the SPGE and SEIR scans were processed by using the mrQ software package (https://github.com/mezera/mrQ) in MATLAB to produce the evaluation of MTV and quantitative T1 maps for each participant. The RF coil bias was corrected by combining SPGE scans with a set of low-resolution unbiased SEIR-EPI scans, producing accurate proton density (PD) and unbiased T1 fits across the brain. The MTV maps were estimated by calculating the fraction of non-water volume in each voxel while CSF voxels were approximated as entirely filled with water[1]. The synthetic T1w images which were spatially matched with MTV maps and optimized for segmentation of brain tissues, were also computed by using mrQ package and then processed using Freesurfer for ROI analysis of qMRI modality (see **Surface-based morphometry analysis**).

ROI analysis for qMRI data.

ROIs were segmented and extracted from Freesurfer's standard pipeline for each participant and saved as volumetric binary masks. All of these masks were generated in native MTV space and matched well with participants' synthetic T1w images through manual inspection. The individual ROI masks were then applied on corresponding MTV and T1 maps for each participant, and averaged MTV and T1 values across voxels within each ROI were computed.

Correlation analysis of qMRI measurements.

Regions with group differences in MTV/T1 values were included for this analysis. The MTV/T1 values within each region were respectively correlated with its GMV and FA values across the two groups. Briefly, each region was defined as a ROI using a volumetric 1-mm-diameter sphere centered on its peak MNI coordinates with the strongest statistical significance based on the results of VBM analysis. To extract FA values within each ROI, FA maps of all participants were aligned onto the FMRIB58_FA_1mm template in standard space by using nonlinear registration[16]. The sphere ROI masks were then applied to the normalized FA maps for all participants. The FA values across voxels within each sphere ROI were averaged and extracted. The GMV values within each ROI were averaged and extracted from the preprocessed GM volumes generated from the VBM analysis for all participants. The Pearson's correlation coefficient (r) and p value between the MTV/T1 and GMV and FA values for each ROI were calculated. The statistical significance was set at $p < 0.05$, FWE corrected.

We also correlated qMRI measurements with HAMD and HAMA scores to test the influence of anxiety on microstructural alternations in MDD. Because that data of some MDD patients were missing, the correlation analysis was only conducted based on the existing scale data (n = 36). HAMA and HAMD scores of each MDD patient nearest to the time of MRI scanning were selected. The demographic data (age, gender and education years) of all MDD patients were included as covariates.

Supplementary structural network analysis

A supplementary structural network analysis was performed to evaluate the effect of participants' demographic data on our dMRI results. The procedures performed in the supplementary analysis were similar to the structural network analysis mentioned above (see **Network construction**) but the effect of participants' age, gender and education years were removed before two-sample *t*-test.

Supplementary VBM analysis

Since there were significant differences in education years between the two groups, we additionally conducted a supplemental VBM analysis to remove the effect of participants' demographic data on the GMV/WMV measures such that further validate the outcome of our study. The procedures of the supplemental VBM analysis were similar to those mentioned above (see **Voxel-based morphometry analysis**) but participants' demographic data (age, gender and education years) were classified as covariates and subsequently regressed out.

Supplementary Results

Alternations of the thalamus-centered structural connectivity in MDD patients

To identify specific pattern of thalamic structural connectivity in MDD patients, we extracted the bilateral thalamus-to-whole-brain connections identified by the NBS analysis (Supplementary Figure 1A). These connections formed a thalamus-centered network, primarily involving the bilateral supplementary motor area, dorsolateral superior frontal gyrus, putamen, precuneus, superior parietal gyrus, superior occipital gyrus and middle frontal gyrus. Specifically, one network centered on the left thalamus (THA.L) is comprised of 15 increased connections with 15 nodes, mainly involving the left supplementary motor area (SMA.L), left paracentral lobule (PCL.L), right putamen (PUT.R), SFGdor.L, SPG.R and left precuneus (PCUN.L). The other network centered on the right thalamus (THA.R) is comprised of 20 edges connecting to 20 nodes, including 16 increased connections and 4 decreased connections. The increased connections linked the THA.R with the bilateral supplementary motor area, right superior occipital gyrus (SOG.R), right postcentral gyrus (PoCG.R), right precentral gyrus (PreCG.R) and left putamen (PUT.L) whereas the decreased connections linked the THA.R with the right parahippocampal gyrus (PHG.R), left caudate (CAU.L), median cingulate and paracingulate gyri (DCG.R) and right pallidum (PAL.R). These connections remained significant at higher supra-threshold of $t > 3.0$. The results of the thalamus-whole brain fiber tracking showed a significant hyper-connectivity pattern in MDD patients compared with HCs after multiple comparison correction (Supplementary Figure 1B). We found that the target regions structurally connecting to the thalamus were located in the cortical regions e.g., the bilateral middle frontal gyrus, middle temporal gyrus, dorsolateral superior frontal gyrus, precentral and postcentral gyrus, precuneus, inferior temporal gyrus, superior temporal gyrus, superior medial frontal gyrus, insula, inferior orbitofrontal gyrus and anterior cingulate and paracingulate gyri as well as subcortical regions including the bilateral putamen,

hippocampus, parahippocampal gyrus, caudate and pallidum. No significant decreased structural connectivity was survived after multiple comparison correction in MDD patients relative to HCs.

The correlation of qMRI measurements across 2 groups

The correlation analysis showed that T1s were negatively correlated with GMV values in the THA.L (r = -0.349, p = 0.002; Supplementary Figure 2) across two groups. There was no significant correlation observed between T1s of the THA.L with HAMA (r = -0.259, p = 0.127) or HAMD scores (r = -0.069, p = 0.689) in MDD patients.

Results of supplementary structural network analysis

By using the NBS method, we only identified a single network after correcting for all the covariates (Supplementary Figure 3). The identified network consisted of 212 edges connecting to 75 nodes, representing increased structural connectivity in MDD patients (Supplementary Figure 3A). Among these nodes, the bilateral putamen, thalamus, dorsolateral superior frontal gyrus, hippocampus, superior parietal gyrus, right inferior temporal gyrus (ITG.R), right caudate (CAU.R), SMA.L, SOG.R, PCUN.L and right middle temporal gyrus (MTG.R) exhibited ten or more increased inter-regional connections, which were consistent with the results in the main text. Similarly, the identified increased connectivity was further classified into the seven-network parcellation with 100 parcels[17]. We found that the increased connections were still predominantly within the subcortical regions and between the subcortical network and another cortical networks. Specifically, the increased connections within the subcortical regions were observed between the PUT.R and the left hippocampus (HIP.L; t = 3.37), the PUT.R and the THA.L (t = 3.26), the PUT.L and the right hippocampus (HIP.R; t = 3.26), the PUT.L and the PUT.R (t = 3.10), the PUT.L and the PAL.R (t = 3.08), the PUT.L and the THA.R (t = 2.94), the HIP.R and the left amygdala (AMYG.L; t = 2.93), the AMYG.L and the CAU.L (t = 2.78) and the HIP.R and the THA.L (t = 2.55). The between-network connections were mainly detected between the subcortical regions and regions within the somatomotor network, specifically between the PUT.L and the left precentral gyrus (PreCG.L; t = 3.50), the PUT.L and the PCL.L (t = 3.49), the THA.L and the PCL.L (t = 3.23), the PUT.R and the PoCG.R (t = 3.08), the PUT.L and the left postcentral gyrus (PoCG.L; t = 3.08), the CAU.L and the right superior temporal gyrus (STG.R; t = 3.02) and the PUT.R and the PreCG.R (t = 3.00). These supplementary results revealed a similar increased connectivity pattern with those in the main text in MDD patients, both indicating the subcortical regions may be closely associated with disrupted structural connectivity in MDD patients. The supplementary results suggest altered structural connectivity in MDD patients is unlikely to be effect by participants' age, gender and education years.

Results of supplementary VBM analysis

The results of the supplementary VBM analysis were comparable to those in the main text (Supplementary Figure 4 and Table 3). A total of 23 clusters showing significant reduced GMV were

identified in MDD patients relative to HCs, while WMV reductions were found in eight clusters (Supplementary Figure 4A). Additionally, the reduced GMV values of the key regions e.g., the bilateral thalamus, bilateral insula, left parahippocampal gyrus (PHG.L), right amygdala (AMYG.R), left inferior orbitofrontal gyrus (ORBinf.L) and ORBsup.L were still observed even participants' demographic data were regressed out. The GMV values of these key regions were also extracted from the identified clusters for visualization (Supplementary Figure 4B). The results of the supplementary VBM analysis indicate that the overall volume reduction in MDD patients is unlikely to be induced by participants' demographic data. Notably, there was still no significant cluster showing larger GMV/WMV in MDD patients compared with HCs.

Supplementary Reference

1. Menon V. Large-scale brain networks and psychopathology: a unifying triple network model. *Trends Cogn Sci*. 2011;15(10):483-506.
2. Mezer A, Yeatman JD, Stikov N, et al. Quantifying the local tissue volume and composition in individual brains with magnetic resonance imaging. *Nat Med*. 2013;19(12):1667-1672.
3. Smith SM, Jenkinson M, Woolrich MW, et al. Advances in functional and structural MR image analysis and implementation as FSL. *Neuroimage*. 2004;23:S208-S219.
4. Jenkinson M, Beckmann CF, Behrens TEJ, Woolrich MW, Smith SM. Fsl. *Neuroimage*. 2012;62(2):782-790.
5. Cui Z, Zhong S, Xu P, Gong G, He Y. PANDA: a pipeline toolbox for analyzing brain diffusion images. *Front Hum Neurosci*. 2013;7:42.
6. Behrens TEJ, Berg HJ, Jbabdi S, Rushworth MFS, Woolrich MW. Probabilistic diffusion tractography with multiple fibre orientations: What can we gain? *Neuroimage*. 2007;34(1):144-155.
7. Gong G, He Y, Concha L, et al. Mapping anatomical connectivity patterns of human cerebral cortex using in vivo diffusion tensor imaging tractography. *Cereb Cortex*. 2009;19(3):524-536.
8. Hagmann P, Cammoun L, Gigandet X, et al. Mapping the structural core of human cerebral cortex. *PLoS Biol*. 2008;6(7):e159.
9. Behrens TEJ, Woolrich MW, Jenkinson M, et al. Characterization and propagation of uncertainty in diffusion-weighted MR imaging. *Magn Reson Med An Off J Int Soc Magn Reson Med*. 2003;50(5):1077-1088.
10. Cao Q, Shu N, An L, et al. Probabilistic diffusion tractography and graph theory analysis reveal abnormal white matter structural connectivity networks in drug-naive boys with attention deficit/hyperactivity disorder. *J Neurosci*. 2013;33(26):10676-10687.
11. Korgaonkar MS, Fornito A, Williams LM, Grieve SM. Abnormal structural networks characterize major depressive disorder: a connectome analysis. *Biol Psychiatry*. 2014;76(7):567-574.
12. Xia M, Wang J, He Y. BrainNet Viewer: a network visualization tool for human brain connectomics. *PLoS One*. 2013;8(7):e68910.
13. Smith SM, Nichols TE. Threshold-free cluster enhancement: addressing problems of smoothing, threshold dependence and localisation in cluster inference. *Neuroimage*. 2009;44(1):83-98.
14. Destrieux C, Fischl B, Dale A, Halgren E. Automatic parcellation of human cortical gyri and sulci using standard anatomical nomenclature. *Neuroimage*. 2010;53(1):1-15.
15. Desikan RS, Ségonne F, Fischl B, et al. An automated labeling system for subdividing the human cerebral cortex on MRI scans into gyral based regions of interest. *Neuroimage*. 2006;31(3):968-980.
16. Smith SM, Jenkinson M, Johansen-Berg H, et al. Tract-based spatial statistics: voxelwise analysis of multi-subject diffusion data. *Neuroimage*. 2006;31(4):1487-1505.
17. Schaefer A, Kong R, Gordon EM, et al. Local-global parcellation of the human cerebral cortex from intrinsic functional connectivity MRI. *Cereb Cortex*. 2018;28(9):3095-3114.

Supplementary Table 1. Abbreviations of regions from AAL atlas.

Index	Regions	Abbreviation
1	Precentral gyrus	PreCG.L
2	Precentral gyrus	PreCG.R
3	Superior frontal gyrus, dorsolateral	SFGdor.L
4	Superior frontal gyrus, dorsolateral	SFGdor.R
5	Superior frontal gyrus, orbital part	ORBsup.L
6	Superior frontal gyrus, orbital part	ORBsup.R
7	Middle frontal gyrus	MFG.L
8	Middle frontal gyrus	MFG.R
9	Middle frontal gyrus, orbital part	ORBmid.L
10	Middle frontal gyrus, orbital part	ORBmid.R
11	Inferior frontal gyrus, opercular part	IFGoperc.L
12	Inferior frontal gyrus, opercular part	IFGoperc.R
13	Inferior frontal gyrus, triangular part	IFGtriang.L
14	Inferior frontal gyrus, triangular part	IFGtriang.R
15	Inferior frontal gyrus, orbital part	ORBinf.L
16	Inferior frontal gyrus, orbital part	ORBinf.R
17	Rolandic operculum	ROL.L
18	Rolandic operculum	ROL.R
19	Supplementary motor area	SMA.L
20	Supplementary motor area	SMA.R
21	Olfactory cortex	OLF.L
22	Olfactory cortex	OLF.R
23	Superior frontal gyrus, medial	SFGmed.L
24	Superior frontal gyrus, medial	SFGmed.R
25	Superior frontal gyrus, medial orbital	ORBsupmed.L
26	Superior frontal gyrus, medial orbital	ORBsupmed.R
27	Gyrus rectus	REC.L
28	Gyrus rectus	REC.R
29	Insula	INS.L
30	Insula	INS.R
31	Anterior cingulate and paracingulate gyri	ACG.L
32	Anterior cingulate and paracingulate gyri	ACG.R
33	Median cingulate and paracingulate gyri	DCG.L
34	Median cingulate and paracingulate gyri	DCG.R
35	Posterior cingulate gyrus	PCG.L
36	Posterior cingulate gyrus	PCG.R
37	Hippocampus	HIP.L
38	Hippocampus	HIP.R
39	Parahippocampal gyrus	PHG.L
40	Parahippocampal gyrus	PHG.R
41	Amygdala	AMYG.L
42	Amygdala	AMYG.R

43	Calcarine fissure and surrounding cortex	CAL.L
44	Calcarine fissure and surrounding cortex	CAL.R
45	Cuneus	CUN.L
46	Cuneus	CUN.R
47	Lingual gyrus	LING.L
48	Lingual gyrus	LING.R
49	Superior occipital gyrus	SOG.L
50	Superior occipital gyrus	SOG.R
51	Middle occipital gyrus	MOG.L
52	Middle occipital gyrus	MOG.R
53	Inferior occipital gyrus	IOG.L
54	Inferior occipital gyrus	IOG.R
55	Fusiform gyrus	FFG.L
56	Fusiform gyrus	FFG.R
57	Postcentral gyrus	PoCG.L
58	Postcentral gyrus	PoCG.R
59	Superior parietal gyrus	SPG.L
60	Superior parietal gyrus	SPG.R
61	Inferior parietal, but supramarginal and angular gyri	IPL.L
62	Inferior parietal, but supramarginal and angular gyri	IPL.R
63	Supramarginal gyrus	SMG.L
64	Supramarginal gyrus	SMG.R
65	Angular gyrus	ANG.L
66	Angular gyrus	ANG.R
67	Precuneus	PCUN.L
68	Precuneus	PCUN.R
69	Paracentral lobule	PCL.L
70	Paracentral lobule	PCL.R
71	Caudate nucleus	CAU.L
72	Caudate nucleus	CAU.R
73	Lenticular nucleus, putamen	PUT.L
74	Lenticular nucleus, putamen	PUT.R
75	Lenticular nucleus, pallidum	PAL.L
76	Lenticular nucleus, pallidum	PAL.R
77	Thalamus	THA.L
78	Thalamus	THA.R
79	Heschl gyrus	HES.L
80	Heschl gyrus	HES.R
81	Superior temporal gyrus	STG.L
82	Superior temporal gyrus	STG.R
83	Temporal pole: superior temporal gyrus	TPOsup.L
84	Temporal pole: superior temporal gyrus	TPOsup.R
85	Middle temporal gyrus	MTG.L
86	Middle temporal gyrus	MTG.R

87	Temporal pole: middle temporal gyrus	TPOmid.L
88	Temporal pole: middle temporal gyrus	TPOmid.R
89	Inferior temporal gyrus	ITG.L
90	Inferior temporal gyrus	ITG.R

The brain regions were defined by Tzourio-Mazoyer et al. (2002)

Supplementary Table 2. Regions with cross-modality structural alternations in MDD patients.

**Regions**	**Structural connectivity [a]**		**Morphometry measurements [b]**			
	Contrast	N	VBM	*p* value	SBM	*p* value
**Cortical regions**						
ORBmid.L	MDD > HC	2	GMV	0.017	Cortical thickness	0.0002
	MDD < HC	1				
**Subcortical regions**						
PUT.L	MDD > HC	27	GMV	0.006	-	
THA.R	MDD > HC	16	GMV	0.005	-	
	MDD < HC	4	WMV	0.004	-	
THA.L	MDD > HC	15	GMV	<0.001	-	
			WMV	0.006	-	
AMYG.R	MDD > HC	1	GMV	0.001	-	

Note: [a] Structural connectivity corresponds to the results of the NBS analysis in diffusion MRI data analysis. [b] Significant group differences were only detected in the contrast of MDD < HC in the results of VBM and SBM analysis. N, the number of connections; VBM, Voxel-based morphometry; SBM, Surface-based morphometry; MDD, major depressive disorder; HC, healthy control. For full names of these abbreviations from AAL atlas, see Supplementary Table 1.

Supplementary Table 3. Clusters showed significant gray matter volume/white matter volume differences between MDD patients and HCs after controlling for the demographic data.

**Anatomical region [a]**	**Side**	**MNI coordinates [b]**			**Cluster size**	***p* value**
		x	y	z		
**GMV**						
THA	L	-8	-10	6	785	<0.001
PHG	L	-14	-34	-9	410	0.001
AMYG	R	22	2	-15	313	0.002
THA	R	12	-9	9	241	0.002
OLF	L	0	8	-8	400	0.003
STG	R	63	-16	10	2075	0.005
INS	R	40	10	0	1912	0.005
INS	L	-32	24	-2	1388	0.005
ORBinf	L	-24	20	-15	1228	0.006
STG	L	-51	-3	0	1088	0.006
REC	R	3	39	-23	726	0.006
TPOsup	L	-22	5	-20	356	0.006

TPOsup	R	57	3	1	265	0.006
ORBsup	L	-24	17	-12	190	0.006
IFGoperc	L	-56	11	6	485	0.007
REC	L	0	56	-20	1057	0.008
IFGtriang	L	-51	17	0	775	0.009
PoCG	L	-26	-46	55	325	0.019
IPL	L	-33	-51	54	1133	0.021
FFG	R	42	-16	-30	276	0.027
SMG	L	-62	-34	39	119	0.030
MFG	L	-24	41	28	300	0.032
SFGdor	L	-16	50	34	229	0.033
**WMV**						
PreCG	R	18	-24	67	1178	0.004
THA	L	-6	-15	10	360	0.004
THA	R	8	-18	12	484	0.006
PoCG	L	-26	-40	49	582	0.008
STG	R	44	-31	3	414	0.009
PreCG	L	-28	-22	55	577	0.011
PCL	R	6	-31	61	310	0.013
DCG	L	-14	-15	46	225	0.020

Note: [a] For full names of these abbreviations from AAL atlas, see Supplementary Table 1. [b] The MNI coordinates of the voxel with maximum statistical significance for identified clusters. Each cluster was represented by a coordinate of the peak voxel in this cluster. GMV, gray matter volume; WMV, white matter volume; MDD, major depressive disorder; HC, healthy control; L, left; R, right.

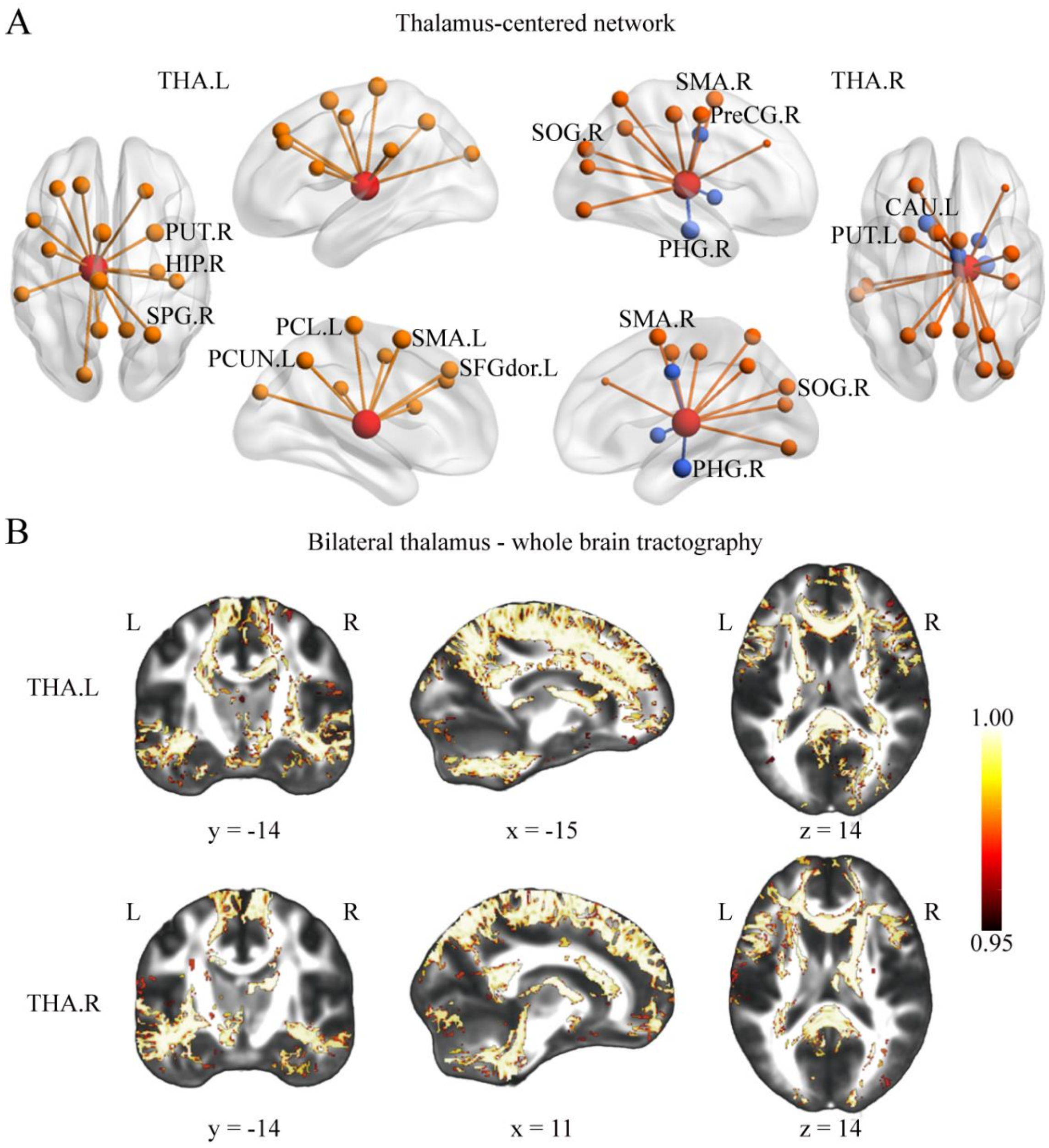

Supplementary Figure 1. Significant altered structural connectivity and connectivity patterns in the thalamus-centered network.

(A) Bilateral thalamus-centered networks identified by the whole-brain NBS analysis were additionally extracted and visualized. The nodes with increased or decreased connections are shown in orange or blue, respectively. Size of nodes indicates *t* value of the edges between the thalamus and target nodes. The bilateral thalamus nodes are emphasized in red. The edges are shown in the same color as their connected nodes with the same widths. Nodes with labels are the hub regions in the whole-brain structural connectivity. **(B)** Significant group differences in the bilateral thalamus-whole brain tractography. The color bar indicates 1-p value with significant threshold of 0.95, corresponding to FWE-corrected $p < 0.05$. Representative coronal, sagittal and axial slices of the significant connections were overlaid on a standard fractional anisotropy template from FSL package. L, left hemisphere; R, right hemisphere. For abbreviation and index of the AAL regions, see Supplementary Table 1.

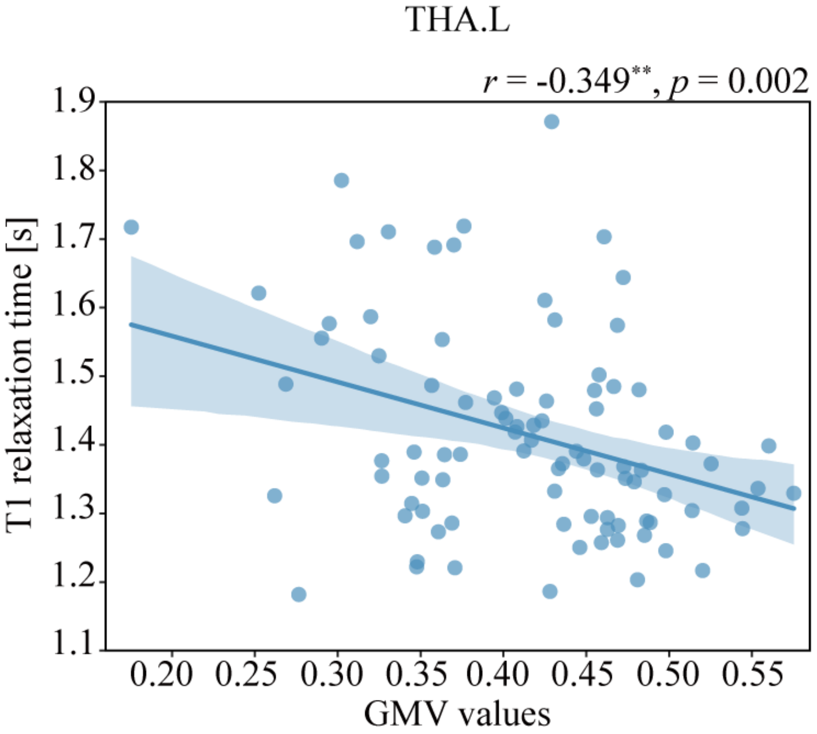

Supplementary Figure 2. Correlation between T1 and gray matter volume values in the left thalamus across 2 groups.

THA.L, the left thalamus; GMV, gray matter volume.

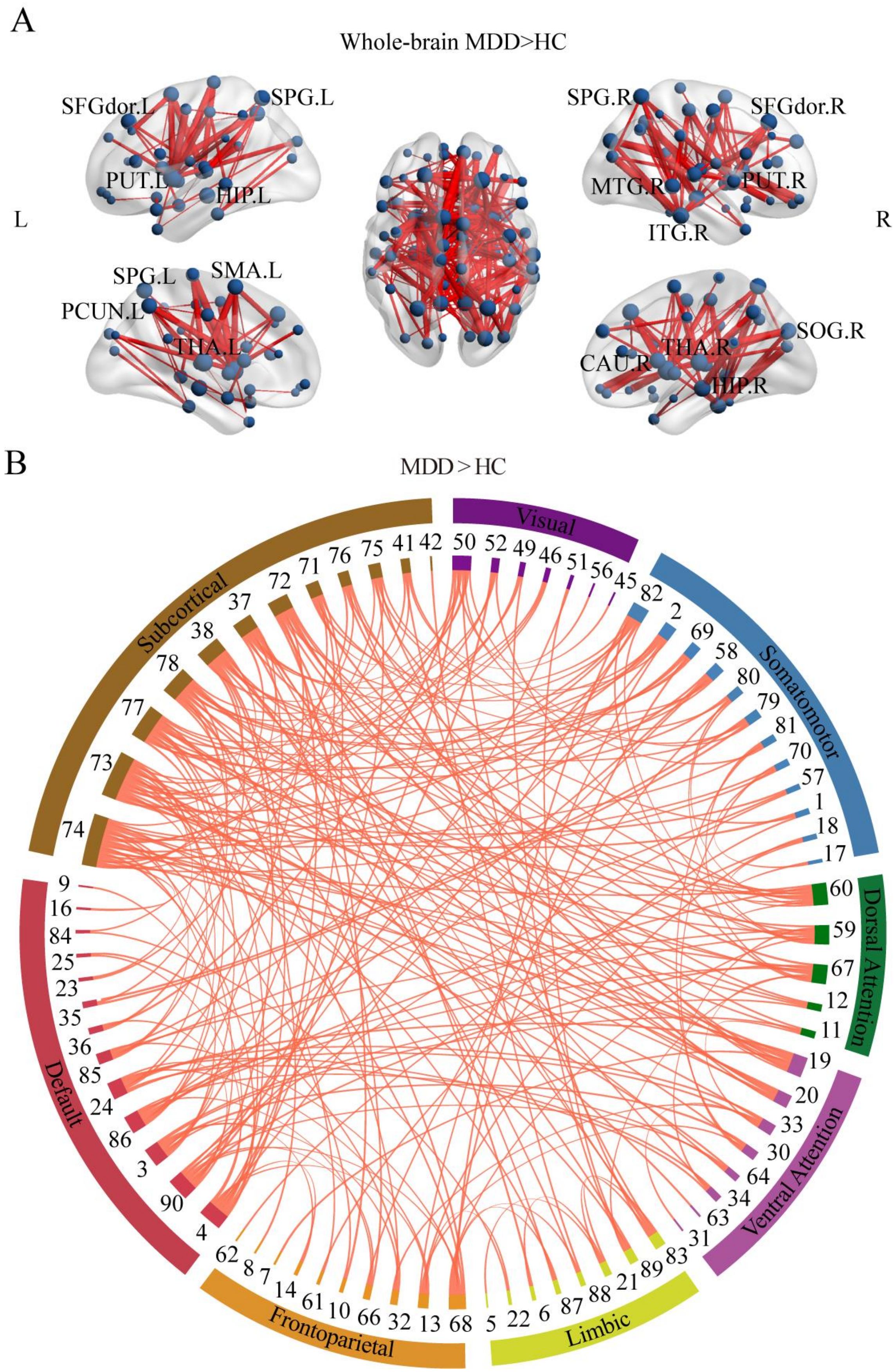

Supplementary Figure 3. Altered structural connectivity in MDD patients with all covariates removed.

(A) Increased structural connectivity identified by the NBS analysis in MDD patients compared with HCs. Nodes with ≥ 10 inter-regional connections are labeled. **(B)** The circle plot depicts the increased connectivity pattern within and between 7 network and subcortical networks. The AAL regions are labeled as their index in the AAL atlas. MDD, major depressive disorder; HC, healthy controls. For abbreviation and index of AAL regions, see Supplementary Table 1.

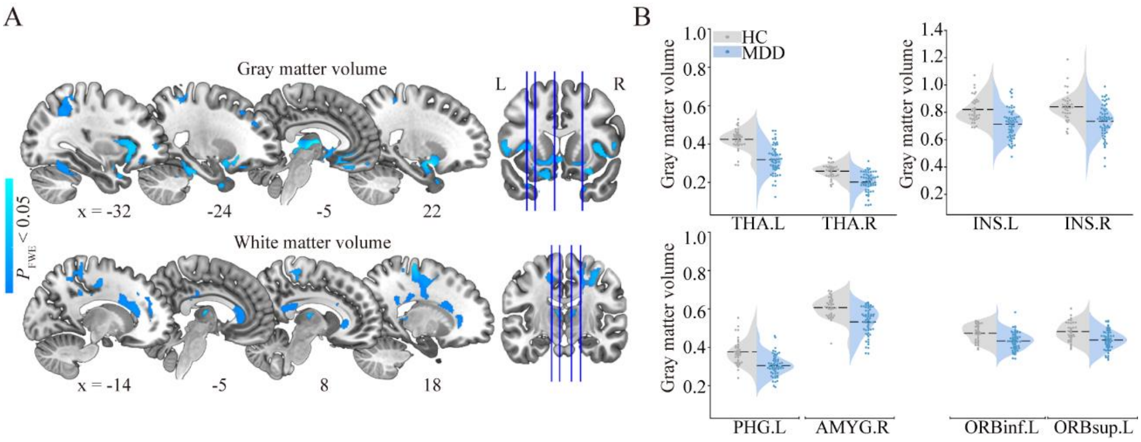

Supplementary Figure 4. Between-group differences in gray and white matter volume with all covariates removed.

(A) The gray matter volume (GMV; upper) and white matter volume (lower) reductions in MDD patients compared with HCs. The color bar indicates *p* value. **(B)** The violin plots depict between-group differences of the GMV values within 8 key regions. Because the raw statistic map was transformed into TFCE values, the GMV values of these regions were extracted from the voxel-level statistic map obtained by using two-sample *t*-test with the threshold of $p < 0.001$ (uncorrected). MDD, major depressive disorder; HC, healthy controls. For abbreviation of AAL regions, see Supplementary Table 1.
